# Robotic gastrectomy for gastric cancer: Current evidence

**DOI:** 10.1002/ags3.12020

**Published:** 2017-07-28

**Authors:** Rana M. Alhossaini, Abdulaziz A. Altamran, Won Jun Seo, Woo Jin Hyung

**Affiliations:** ^1^ Department of Surgery Yonsei University College of Medicine Seoul Korea; ^2^ Gastric Cancer Center Yonsei Cancer Center Yonsei University Health System Seoul Korea; ^3^ Robot and MIS Center Severance Hospital Yonsei University Health System Seoul Korea

**Keywords:** gastrectomy, gastric cancer, laparoscopic surgery, minimally invasive surgery, robotic surgery

## Abstract

The robotic system has gained wide acceptance in specialties such as urological and gynecological surgery. It has also been applied in the field of upper gastrointestinal surgery. Since the first implementation of the robotic system for the treatment of gastric adenocarcinoma, the procedure has been found to be safe and feasible. Although robotic gastrectomy does not meet our expectations and yield better results than laparoscopic gastrectomy, this procedure seems to provide several advantages over laparoscopy such as reduced blood loss, shorter learning curves and increased number of retrieved lymph nodes. However, as many case series, including a recent multicenter study, have revealed, higher cost and longer operation time are the major limitations of robotic gastrectomy. Furthermore, there are no results from well‐designed randomized clinical trials comparing the two procedures. New procedures in much more technically demanding cases will test the genuine benefits of robotic gastrectomy.

## 1 INTRODUCTION

Minimally invasive surgical approaches to gastric cancer have been used as a tool to improve postoperative outcomes in patients undergoing gastrectomy for gastric cancer.^1^ Improved postoperative outcomes for patients include reduced pain, lower risk of complications, less blood loss, shorter hospital stay, and faster return to normal activities. Since robotic surgery was first introduced in the late 1990s, wide application and accumulation of experience have continued.^2^, ^3^ Moreover, it has overcome some of the limitations of conventional laparoscopy by providing increased accuracy of tremor filtered and wristed instrumental movements, along with seven degrees of freedom and the ability to scale motions.^4^, ^5^

Many experienced laparoscopic surgeons have adopted robotic surgery for the treatment of gastric cancer. Within a decade after the initial reports describing the use of robots for the treatment of early‐stage gastric cancer, robotic gastrectomy has been found to be a safe and feasible alternative to conventional laparoscopic gastrectomy.^6^, ^7^ However, critical issues such as cost‐effectiveness and oncological safety for advanced cancer remain to be solved to expand the indications for robotic gastrectomy for gastric cancer. In the present review, we discuss the current evidence for the use of robotic gastrectomy, including indications and applications, perioperative outcomes, cost, learning curve, oncological outcomes, and future perspectives.

## 2 INDICATIONS OF ROBOTIC APPLICATION

The indications for robotic gastrectomy are same as those of laparoscopic gastrectomy for gastric cancer. Initial indications for robotic gastrectomy were early gastric cancers without evidence of lymph node metastasis based on clinical diagnosis. It was expanded to include clinical stage T1‐2 cancers with or without perigastric lymph node metastasis, except for lesions for which endoscopic submucosal dissection (ESD) was indicated.^8^, ^9^ So far, in Korea and Japan, neither robotic nor laparoscopic gastrectomy is indicated for the treatment of serosa‐involved advanced gastric cancer.^10^ As there are some reports on serosa‐involved gastric cancer in the use of robotics or laparoscopy, it seems that it would not be a clear contraindication to a minimally invasive approach. However, there are limitations to a minimally invasive approach such as bulky tumors, massive lymphadenopathy or tumors that require multi‐organ resection.^11^

To determine the proximal resection line for R0 resection, preoperative endoscopic placement of radiopaque hemoclips or intraoperative endoscopic localization is required, especially for small or non‐palpable tumors.^12^, ^13^, ^14^, ^15^ Regarding the extent of lymph node dissection, it follows the Japanese classification of Gastric Carcinoma guidelines: D1+ lymph node dissection is indicated for clinically early gastric cancer without evidence of lymph node metastasis and D2 is indicated for advanced gastric cancer or any evidence of regional lymph node involvement.^10^

## 3 PERIOPERATIVE OUTCOMES

### 3.1 Operative time

Overall, several reports documented that operative time was significantly longer for the robotic procedure ranging from 202 to 439 minutes compared with 171 to 361 minutes in laparoscopy^6^, ^7^, ^16^ (Table 1). Increase in operative time was initially attributed to the docking time, but this no longer seems to be a contributing factor.^15^ Moreover, the operative time gradually decreased with the accumulation of surgical experience in robotic gastrectomy. Nonetheless, longer operative time is regarded as the main drawback of robotic gastrectomy, because it may affect patient recovery, especially in those with comorbidity.^32^, ^33^, ^34^

**Table 1:** Surgical and pathological outcomes of robotic vs laparoscopic gastrectomy techniques for gastric cancer treatment

Author	Year	Type of approach	No. patients	Operation time (min)	Blood loss (mL)	No. retrieved LN
Pugliese et al.^17^	2009	R	9	350	92	27.5
		L	46	236	156	31.5
Song et al.^18^	2009	R, initial	20	230	94.8	35.3
		L, initial	20	289	‐	31.5
		L, recent	20	134	39.5	42.7
Pugliese et al.^16^	2010	R	16	344	90	25
		L	48	235	148	31
Woo et al.^6^	2011	R	236	219.5	91.6	39.0
		L	591	170.7	147.9	37.4
Yoon et al.^19^	2012	R	36	305.8	‐	42.8
		L	65	210.2	‐	39.4
Eom et al.^20^	2012	R	30	229.1	152.8	30.2
		L	62	189.4	88.3	33.4
Kang et al.^21^	2012	R	100	202	93.2	‐
		L	282	173	173.4	‐
Hyun et al.^22^	2013	R	38	234.4	131.3	32.8
		L	83	220.0	130.4	32.6
Huang et al.^23^	2014	R	72	357.9	79.6	30.6
		L	73	319.8	116.0	28.1
Junfeng et al.^24^	2014	R	120	234.8	118.3	34.6
		L	394	221.3	137.6	32.7
Son et al.^7^	2014	R	51	264.1	163.4	47.2
		L	58	210.3	210.7	42.8
Noshiro et al.^25^	2014	R	21	439	96	44
		L	160	315	115	40
Lee et al.^26^	2015	R	133	217.5	47	41.2
		L	267	171	87.1	39.9
Park et al.^27^	2015	R	148	254.5	171.3	46.5
		L	622	188.5	145.5	38.8
Suda et al.^28^	2015	R	88	381	46	40
		L	438	361	34	38
Kim et al.^29^, ^a^	2016	R	223	226	50	33
		L	211	180	60	32
Shen et al.^30^	2016	R	93	257.1	176.6	33
		L	330	226.2	212.5	31.3
Kim et al.^31^	2016	R	87	248.4	‐	37.1
		L	288	230	‐	34.1

a Prospective study.

L, laparoscopic; LN, lymph nodes; R, robotic; ‐, no data.

### 3.2 Blood loss

Many reports comparing robotic and laparoscopic gastrectomy revealed that there is significant reduction in blood loss with a range of 46–176mL with robotic approach and 34–212mL with laparoscopic approach^6^, ^7^, ^16^ (Table 1). Meanwhile, data from a recent multicenter non‐randomized comparative trial showed that there was no difference in estimated blood loss between robotic and laparoscopic gastrectomy.^29^ However, this should be further scrutinized, because there might be a certain group of patients (ie patients with higher body mass index or those who underwent extensive lymph node dissection) that benefit from the robotic procedure with less blood loss. The reduced blood loss may result from robotic system advantages such as the three‐dimensional (3D) view and the tremor‐filtered articulated function that help in better detection of vessels and facilitate control of intra‐abdominal bleeding. In general, reduced blood loss may have less effect on the short‐term clinical course, although it is statistically significant. However, it may have an effect on long‐term oncological outcomes, especially in advanced gastric cancer, as shown in the literature.^25^, ^28^

### 3.3 Complications

Most publications comparing robotic with laparoscopic gastrectomy demonstrated similar complication rates (Table 2). A recent multicenter prospective comparative study showed similar overall complication rates of 11.9% in the robotic group and 10.3% in the laparoscopic group and the rate of major complications of 1.1% in both groups.^29^ So far, one publication has shown significantly fewer complications for robotic gastrectomy in comparison to laparoscopic gastrectomy regarding postoperative pancreatic fistula at a rate of 2.3% vs 11.4%, respectively. It was suggested that this can be attributed to the integrity of the robot‐specific functions which allow minimal pressure on the pancreas, subsequently leading to less parenchymal injury.^28^

**Table 2:** Postoperative outcomes of robotic vs laparoscopic gastrectomy techniques for gastric cancer treatment

Author	Y	Type of approach	Hospital stay (d)	Morbidity (%)	Mortality (%)
Pugliese et al.^17^	2009	R	11	‐	‐
		L	10	‐	‐
Song et al.^18^	2009	R, initial	5.7	5	0
		L, initial	7.7	5	0
		L, recent	6.2	10	0
Pugliese et al.^16^	2010	R	10	6	‐
		L	10	12.5	‐
Woo et al.^6^	2011	R	7.7	11	0.4
		L	7.0	13.7	0.3
Yoon et al.^19^	2012	R	8.8	16.7	0
		L	10.3	15.4	0
Eom et al.^20^	2012	R	7.9	13	0
		L	7.8	6	0
Kang et al.^21^	2012	R	9.8	14	0
		L	8.1	10.3	0
Hyun et al.^22^	2013	R	10.5	47.3	0
		L	11.9	38.5	0
Huang et al.^23^	2014	R	11.0	12.5	1.4
		L	13.2	8.2	1.4
Junfeng et al.^24^	2014	R	7.8	5.8	‐
		L	7.9	4.3	‐
Son et al.^7^	2014	R	8.6	15.7	2.0
		L	7.9	22.4	0
Noshiro et al.^25^	2014	R	8	9.5	0
		L	13	10.0	0
Lee et al.^26^	2015	R	6.2	10.5	‐
		L	7	12.7	‐
Park et al.^27^	2015	R	7.9	2.8	0
		L	7.9	4.6	0.5
Suda et al.^28^	2015	R	14	2.3	1.1
		L	15	11.4	0.2
Kim et al.^29^, ^a^	2016	R	6	30	0
		L	6	30	0
Shen et al.^30^	2016	R	9.4	9.8	‐
		L	10.6	10	‐
Kim et al.^31^	2016	R	6.7	5.7	1.1
		L	7.4	9	0.3

a Prospective study.

L, laparoscopic; R, robotic; ‐, no data.

### 3.4 Length of hospital stay

Most investigators found no differences in length of hospital stay when they compared robotic with laparoscopic gastrectomy (Table 2). A recent multicenter prospective comparative study also showed no difference in hospital stay between robotic and laparoscopic gastrectomy.^29^ However, a retrospective study demonstrated a reduction in hospital stay between the two approaches with a mean of 14 days in the robotic group and 15 days in the laparoscopic group.^28^ Another comparative study revealed a bigger reduction of hospital stay in distal subtotal gastrectomy. The robotic distal subtotal gastrectomy group showed a mean of 8 days of postoperative hospital stay which was 5 days shorter than that of the laparoscopic gastrectomy group.^25^ However, as most studies were biased mainly as a result of the different groups of patients undergoing each surgical procedure, a well‐designed randomized clinical trial should be done. Based on the current results, robotic gastrectomy does not seem to have advantages over laparoscopic gastrectomy in terms of postoperative recovery.

### 3.5 Cost

Higher cost has been a consistently reported disadvantage of the robotic approach for gastric cancer. A recent prospective study demonstrated that the cost of robotic surgery was US$4490 more than that of laparoscopic surgery. The Korean National Health Insurance System covers perioperative care for both procedures. Although most of the operation costs for laparoscopic gastrectomy are covered by national insurance, none of the operation costs for robotic gastrectomy are covered. Thus, the actual cost charged to patients was US$7326 more for the robotic than for the laparoscopic group.^29^ However, to identify whether the cost of robotic surgery is higher than that of laparoscopic gastrectomy, more comprehensive analyses considering different insurance systems need to be carried out. Nonetheless, more evidence showing the benefits of robotic gastrectomy are needed to justify this high‐cost operation. Furthermore, the number of surgeons involved in the robotic operations is less than laparoscopic surgery, this may be advantageous to lessen the cost of robotic surgery.

### 3.6 Learning curve

One suggested advantage of robotic surgery is its short learning curve compared to laparoscopic surgery. Thus, from the initial experience, robotic surgery can be carried out safely if it is conducted by a surgeon experienced in laparoscopic surgery. The learning curve of robotic gastrectomy demonstrates a quicker adaptation with most studies reporting 11‐25 cases to be sufficient for experienced gastric cancer surgeons,^20^, ^35^, ^36^ whereas 40‐60 cases of surgical experience are required to overcome the learning curves associated with laparoscopic gastrectomy.^37^, ^38^ However, there is no study directly comparing the learning curve effect of robotic surgery in cases of surgeons having no laparoscopic experience. Although it would be ideal to explore a learning curve of robotic gastrectomy in surgeons without experience of laparoscopic gastrectomy, it is almost impossible because of the popularity of laparoscopic gastrectomy in recent years.

## 4 ONCOLOGICAL OUTCOMES

As the use of robotic surgery in gastric surgery is relatively recent, it is a bit early to confirm the long‐term oncological results of robotic gastrectomy compared to laparoscopic gastrectomy. Oncological outcomes other than survival or recurrence, such as the number of lymph nodes harvested and margin status, are reported to be similar between robotic and laparoscopic approaches^30^ (Table 1). However, a potential advantage of robotic surgery was found in carrying out D2 lymphadenectomy. Dissection of lymph nodes around the superior mesenteric vein (station #14), celiac axis (station #9), splenic vessels (station #11), and splenic hilum lymph nodes (station #10), is the most frequent source of intraoperative bleeding because of the anatomical complexity of the vascular structures. This complexity can be overcome by improved imaging with the robotic endo‐wrist movements which provide better access. With these technical superiorities, robotic surgery retrieved more lymph nodes in the extra‐perigastric area.^7^ A case series of 316 robotic gastrectomies was previously reported, 95 of those underwent subtotal gastrectomy with D2 lymph node dissection. A mean of 41.8 lymph nodes (range 11‐89) was retrieved. Overall survival during a mean follow‐up period of 60.5 months was 92.8%.^39^

Another comparative study revealed that the robotic gastrectomy group had a significantly higher number of harvested second‐tier lymph nodes than did the laparoscopic group, whereas first‐tier lymph node number did not differ between the two groups. As for tumor recurrence, it was 4.2% in the robotic and 7.1% in the laparoscopic group during a follow‐up period of 15 and 19 months, respectively.^24^ As for long‐term survival and recurrence, although some studies have shown it was similar for both laparoscopic and robotic surgery, longer follow‐up periods with larger sample size studies that include advanced gastric cancer patients are still required to determine the oncological efficacy of robotic surgery.

## 5 YONSEI EXPERIENCE OF ROBOTIC GASTRECTOMY FOR CANCER

**Figure 1:**
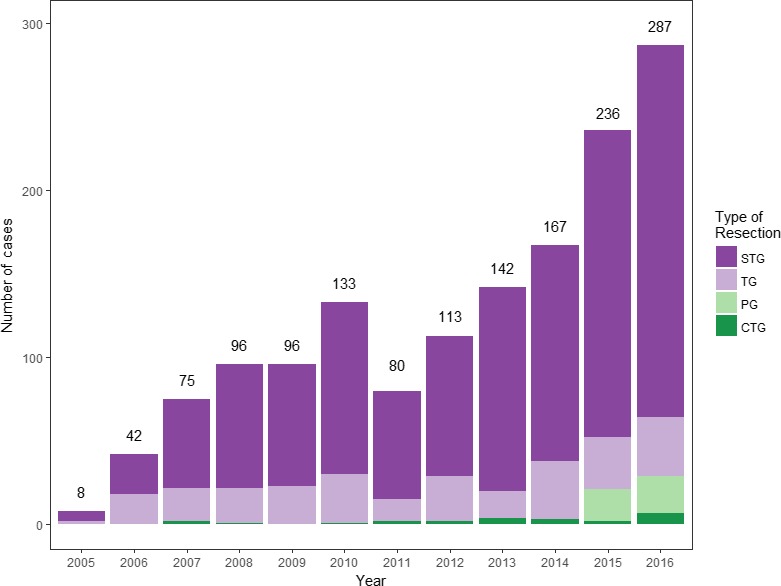
Annual number of robotic gastrectomies carried out for gastric cancer. CTG, completion total gastrectomy; PG, proximal gastrectomy; STG, subtotal gastrectomy; TG, total gastrectomy

Since robotic gastrectomy for gastric cancer was first carried out in 2005 at Severance Hospital, Yonsei University Health System, we have carried out over 1000 robotic gastrectomies, so far. The number of robotic gastrectomies has tended to increase (Figure 1). Initially, relatively early clinical stage gastric cancer patients were indicated for robotic surgery. After achieving more experience with comparable results compared with laparoscopic gastrectomy, we applied robotic surgery to more advanced gastric cancer.

We compared surgical outcomes of our experience of robotic gastrectomy with early (initial 500 robotic gastrectomies) and later (after 500 robotic gastrectomies) robotic gastrectomy carried out between 2005 and 2015 (Table 3). Baseline patient's characteristics, such as age, gender, comorbidities, previous surgical history, BMI, and family history, were not different in both groups. Operation time and blood loss decreased with time, about 14 minutes (*P*<.001) and 16 mL (*P*=.027), respectively. Number of retrieved lymph nodes increased in later cases (*P*<.001). Time to first flatus was statistically different in the two groups (*P*<.001) but initiation of soft diet was not statistically different (*P*=.221). There were four (0.3%) mortalities, two in the initial period and the others in the later period. Overall, results of robotic gastrectomy in our hospital demonstrated acceptable outcomes. Nowadays, robotic gastrectomy is one of the routine procedures for the treatment of gastric cancer in our hospital.

**Table 3:** Comparison of initial and later experience of robotic gastrectomy

	Initial (N=500)	Later (N=687)	*P* value
Age (y, mean±SD)	54.0±12.6	54.1±21.1	>.999
Gender (n, %)			.220
Male	300 (60.0%)	386 (56.1%)	
Female	200 (40.0%)	301 (43.8%)	
ASA score (n,%)			.220
1	155 (31.0%)	177(25.7%)	
2	273 (54.6%)	398 (57.9%)	
3	71 (14.2%)	109 (15.8%)	
4	1 (0.2%)	3 (0.4%)	
BMI (kg/m^2^, mean±SD)	23.5±3.1	23.5±3.2	.937
Type of resection (n,%)			<.001
STG	374 (74.8%)	543 (78.9%)	
TG	121 (24.2%)	114 (16.6%)	
PG	0 (0.0%)	19 (2.8%)	
CTG	5 (1.0%)	12 (1.7%)	
POD (d, mean±SD)	7.3±12.8	6.6±9.7	.337
Blood loss (mL, mean±SD)	76.1±130.0	60.3±91.4	.027
OP time (min, mean±SD)	229.3±55.5	215.7±67.0	<.001
Retrieved LN (mean±SD)	39.4±15.3	44.5±18.9	<.001
Time to flatus (mean±SD)	2.8±0.8	3.3±0.8	<.001
Time to soft diet (mean±SD)	4.8±8.5	4.3±2.4	.221
TNM stage (n, %)^a^			.503
Ia	355 (71%)	468 (68.1%)	
Ib	51 (10.2%)	76 (11.1%)	
IIa	33 (6.6%)	52 (7.6%)	
IIb	21 (4.2%)	40 (5.8%)	
IIIa	15 (3.0%)	19 (2.8%)	
IIIb	13 (2.6%)	18 (2.6%)	
IIIc	9 (1.8%)	13 (1.9%)	
IV	0 (0.0%)	1 (0.1%)	
X	3 (0.6%)	0 (0.0%)	
Complications (n, %)^b^			.001
No	438 (87.6%)	549 (79.9%)	
Yes	62 (12.4%)	138 (20.1%)	

a Based on the 7th edition of the American Joint Committee on Cancer Staging Manual.^40^

b Based on Clavien‐Dindo Surgical Complication Grading System.^41^

ASA, American Society of Anesthesiologists physical status; BMI, body mass index; CTG, completion total gastrectomy; LN, lymph nodes; OP, operation; POD, post operation day; STG, subtotal gastrectomy; TG, total gastrectomy.

## 6 NEW TECHNOLOGIES IN ROBOTIC SURGERY SYSTEM

### 6.1 Newer version system and new instruments

The da Vinci Xi Surgical System (Intuitive Surgical Inc., Sunnyvale, CA, USA) is a relatively new robotic system that is expected to overcome the limitations of the previous platform. Unlike the da Vinci Si Surgical System (Intuitive Surgical Inc., Sunnyvale, CA, USA), the Da Vinci Xi System offers better anatomical access in multi‐quadrant surgical access because of the overhead boom rotation without axis limitation.^42^ It is also equipped with a lighter and smaller endoscope that fits all ports with a highly magnified 3D, high‐definition, end‐mounted camera for improved vision. In addition, Da Vinci Xi has narrower arms and a longer instrument shaft which gives surgeons a better reach.^43^, ^44^ However, to our knowledge, there are currently no studies that have compared the use of Si and Xi systems in gastric surgery. Thus, whether the Xi system is better than the Si system for gastrectomy is not yet explored.

Another new platform is the da Vinci Single Site Surgical System (Intuitive Surgical Inc., Sunnyvale, CA, USA) robot surgery which aims to be even less invasive than the conventional robotic system. It is designed to allow robotic surgery to be carried out in a virtually scarless manner. With the use of a single‐port system, semi‐rigid instruments are used through the curved instrument cannulae to minimize external collision of instrument and camera arms. This new system has overcome the ergonomic disadvantages of conventional single‐port laparoscopic surgery, although current semi‐rigid or wristed devices are not sufficient to carry out complex operations. However, this has the potential to improve the outcomes of reduced or single‐port robotic gastrectomy. However, as it is a relatively new system, this needs further evaluation.^45^

**Figure 2:**
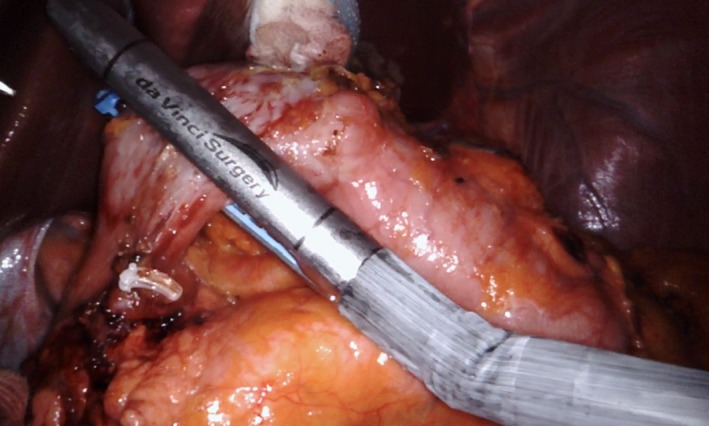
Endowrist stapler (Intuitive Surgical Inc., Sunnyvale, CA, USA) used during gastrectomy

Apart from systemic changes, various instruments were developed, such as stapling devices, vessel sealing system. A linear stapler was incorporated into the robotic system that can be controlled by the surgeon at the console without the help of an assistant (Figure 2). Although this is considered more convenient, a comparative study is warranted to determine its safety and cost efficiency. Introduction of new instruments to the robotic system is presumed to be advantageous as a step further to advance this technology. Possible theoretical new instruments to the robotic system such as multi‐firing clips, angulating ultrasonic sheers, and autonomous knot‐tying could reduce operation time and improve operative outcomes.^46^

### 6.2 Use of near infrared imaging with fluorescent material

**Figure 3:**
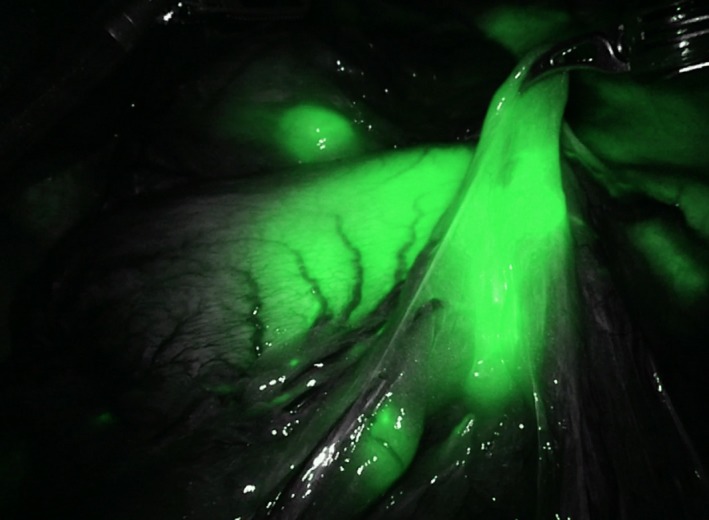
Near infrared imaging after indocyanine green injection identifying tumor location as well as lymphatic drainage

Robotic platform allows the integration of functional imaging such as the use of near infrared imaging after injection of indocyanine green. Near infrared imaging is used as a navigation tool for tumor localization and lymph node dissection (Figure 3). Combined with the robotic visuals in minimally invasive gastric cancer surgery, it has significantly changed the understanding of the pattern of lymphatic drainage, as well as increasing the number of harvested lymph nodes. As proper lymphadenectomy is crucial for an adequate oncological operation for gastric cancer, near infrared imaging improves visualization of vascular anatomy and lymph node identification.^47^ This technology has been incorporated into multiple specialties as well preventing organ injuries such as to the ureter and biliary ducts.^48^ In the field of colorectal surgery, in addition to its use in primary surgery, near infrared imaging has been used as a guide for resection of liver metastasis as well as detection of peritoneal carcinomatosis.^49^ It also aids in identifying the lymphatic drainage pattern of esophageal cancer, which revealed that most distal esophageal and esophagogastric junction tumors drain into the left gastric lymph nodes, whereas other lymph node stations were less commonly involved.^50^

### 6.3 Image‐guided surgery

Obtained intraoperatively or preoperatively, computed tomography and magnetic resonance imaging scans reconstructive images are used as a guide for primary tumor or lymph node dissection by incorporating them into the robotic system.^51^ Image‐guided navigation helps to properly identify stomach vasculature and eliminate the possibility of vascular injuries.^4^, ^52^ These techniques are more advanced in other fields such as neurosurgery and orthopedic surgery. In gastric surgery, however, image‐guided surgery still needs to be explored further in order to gauge the potential advantages.

### 6.4 Intraoperative endoscopy

Endoscopic interventions have evolved significantly in recent years going from a diagnostic tool to an alternative to radical open surgery in early gastric cancer patients who fit the indication for endoscopic mucosal resection.^52^ Furthermore, similar to computed tomography images, real‐time integrated endoscopic images can be visualized using a multiport display program with one‐screen views of the endoscopic procedure during surgery.^53^ This facilitates the performance of intraoperative endoscopies which, in combination with the robotic system, potentially eases tumor localization during surgery.

## 7 CONCLUSION

Robotic technique has been proven to be safe and feasible for use in gastric cancer surgery instead of the laparoscopic technique. Also, with the advancement of robotic technology, treatment could be extended to cover more advanced cases of gastric cancer to improve the quality of lymph node dissection. However, more evidence is needed to determine the actual benefits and cost‐efficiency. In addition, continued development of robotic technology is still required to achieve a better environment for improved surgery and the field of gastric cancer surgery is the exemption.

## DISCLOSURE

Conflict of Interest: The authors declare no conflict of interest.
